# Post-mortem genetic testing in sudden cardiac death and genetic screening of relatives at risk: lessons learned from a Czech pilot multidisciplinary study

**DOI:** 10.1007/s00414-023-03007-z

**Published:** 2023-05-13

**Authors:** Pavel Votýpka, Alice Krebsová, Patricia Norambuena-Poustková, Petra Peldová, Štěpánka Pohlová Kučerová, Markéta Kulvajtová, Petra Dohnalová, Matěj Bílek, Veronika Stufka, Kristina Rücklová, Iva Grossová, Hanka Wünschová, Terezia Tavačová, Jana Hašková, Markéta Segeťová, Jakub Štoček, Andrea Gřegořová, Veronika Zoubková, Jana Petřková, Martin Dobiáš, Michal Makuša, Alžběta Blanková, David Vajtr, Hynek Řehulka, Ivan Šubrt, Alexander Pilin, Petr Tomášek, Jan Janoušek, Josef Kautzner, Milan Macek

**Affiliations:** 1 Department of Biology and Medical Genetics, 2nd Faculty of Medicine, Charles University and Motol University Hospital, V Úvalu 84, 150 06 Prague 5, Czech Republic; 2 Department of Cardiology, Center for Inherited Cardiovascular Diseases, IKEM, Vídeňská 1958/9, 140 21 Prague 4, Czech Republic; 3 Department of Forensic Medicine, Faculty of Medicine in Hradec Králové, Charles University and University Hospital Hradec Králové, Prague, Czech Republic; 4 Institute for Forensic Medicine, 3rd Faculty of Medicine, Charles University, Prague, Czech Republic; 5 Department of Forensic Medicine, Faculty of Medicine, University Hospital Bulovka, Charles University, 2nd Prague, Czech Republic; 6 Institute of Forensic Medicine and Toxicology, 1st Faculty of Medicine, Charles University, Prague, Czech Republic; 7 Paediatric Department, 3rd Faculty of Medicine, Charles University, Prague, Czech Republic; 8 Forensic Department of Military University Hospital, Prague, Czech Republic; 9 Faculty of Medicine, Children’s Heart Centre, Charles University and Motol University Hospital, 2nd Prague, Czech Republic; 10 Department of Biology and Medical Genetics, University Hospital Ostrava, Ostrava, Czech Republic; 11 1st Department of Internal Medicine - Cardiology and Laboratory of Cardiogenomics, University Hospital Olomouc and Palacky University, Olomouc, Czech Republic; 12 Institute of Medical Genetics, University Hospital Olomouc and Palacky University, Olomouc, Czech Republic; 13 Institute of Pathological Physiology, University Hospital Olomouc and Palacky University, Olomouc, Czech Republic; 14 Institute of Forensic Science and Medical Law, University Hospital Olomouc and Palacký University, Olomouc, Czech Republic; 15 Forensic Department, Hospital České Budějovice, České Budějovice, Czech Republic; 16 Department of Forensic Medicine and Toxicology, Liberec Regional Hospital, Liberec, Czech Republic; 17 Institute of Forensic Medicine, Faculty of Medicine in Pilsen, Charles University, Prague, Czech Republic; 18 Department of Medical Genetics, University Hospital Pilsen, Pilsen, Czech Republic

**Keywords:** Sudden cardiovascular death, Molecular autopsy, Forensic genetics, Sudden death prevention, Inherited cardiovascular diseases

## Abstract

Sudden cardiac death (SCD) might have an inherited cardiac condition background. Genetic testing supports post-mortem diagnosis and screening of relatives at risk. Our aim is to determine the feasibility of a Czech national collaboration group and to establish the clinical importance of molecular autopsy and family screening. From 2016 to 2021, we have evaluated 100 unrelated SCD cases (71.0% males, age: 33.3 (12.8) years). Genetic testing was performed by next-generation sequencing utilizing a panel of 100 genes related to inherited cardiac/aortic conditions and/or whole exome sequencing. According to autopsy, cases were divided into cardiomyopathies, sudden arrhythmic death syndrome, sudden unexplained death syndrome, and sudden aortic death. We identified pathogenic/likely pathogenic variants following ACMG/AMP recommendations in 22/100 (22.0%) of cases. Since poor DNA quality, we have performed indirect DNA testing in affected relatives or in healthy parents reaching a diagnostic genetic yield of 11/24 (45.8%) and 1/10 (10.0%), respectively. Cardiological and genetic screening disclose 83/301 (27.6%) relatives at risk of SCD. Genetic testing in affected relatives as starting material leads to a high diagnostic yield offering a valuable alternative when suitable material is not available. This is the first multidisciplinary/multicenter molecular autopsy study in the Czech Republic which supports the establishment of this type of diagnostic tests. A central coordinator and proper communication among centers are crucial for the success of a collaboration at a national level.

**Supplementary Information:**

The online version contains supplementary material available at 10.1007/s00414-023-03007-z.

## Introduction

Sudden unexplained death (SUD) is defined as an unexplained, unexpected sudden death occurring in an individual older than 1 year. The main cause of SUD is sudden cardiac death which is defined as death occurring within an hour of the onset of symptoms if witnessed or within 24 h from the moment when the decedent was last observed alive without symptoms if unwitnessed [1, 2]. The global annual SCD incidence has been estimated to be 4–5 million cases per year approximately [3]. Approximately 1 to 3 per 100,000 individuals younger than 35 years die suddenly or unexpectedly every year [2]. Coronary artery disease (CAD) is responsible for 80% of SCD cases mainly in the older population. Nevertheless, inherited cardiac conditions including familiar hyperlipoproteinemia causing the premature CAD remain to be the common cause of SCD until 50 years of age also in Czech Republic [3, 4]. Some SCD cases may have a genetic background, mostly with autosomal dominant pattern (50% probability regardless of gender), and there is a significant risk of developing an identical disease with the risk of cardiac arrest in first-degree relatives [5]. Therefore, the post-mortem genetic testing, together with cardiac screening of first-degree relatives, is recommended by European guidelines [6–8]. The scope of examination of survivors at risk was defined in a document of the World Organisation for Heart Rhythm Disorders (APHRS/HRS) and elsewhere [2, 9, 10]. There are recommended autopsy procedures developed within the Association for European Cardiovascular Pathology (AECVP), which aim to standardize the autopsy procedure and diagnostics, incl. spectra of additional laboratory tests at SCD [7, 8].

According to the autopsy results and based on macroscopic and microscopic findings, the categories of SCD types are defined internationally in terms of cardiomyopathy (CM), sudden arrhythmic death syndrome (SADS), and sudden unexplained death in individuals younger or older than 1 year (sudden unexplained death syndrome (SUDS) or sudden unexplained deaths in infant (SUDI)). Sudden unexplained death in epilepsy (SUDEP) is mentioned separately, when epilepsy may be an incorrect diagnosis for unconsciousness due to sustained ventricular arrhythmias, or some epilepsy may be a form of both cerebral and cardiac channelopathies [2, 11].

The AECVP best practices further define cases in which post-mortem genetic testing, sometimes referred to as molecular autopsy, should be performed to pinpoint the cause of SCD and the associated primary prevention of cardiac arrest in relatives [8]. Post-mortem genetic testing of the deceased should be followed, or under ideal conditions, accompanied by clinical genetic counselling and cardiological screening of first-degree relatives [2, 12].

Finding out the causes of SCD therefore represents a multidisciplinary process in which autologous physicians, clinical geneticists, molecular geneticists, cardiologists for children and adults a psychologist, neurologist, lipidologist, general practitioner, and other specialties according to the individual needs of individual cases [13]. Regarding complex issues, this type of diagnostics is concentrated in tertiary care centers.

In the following text, we present the results of a multicenter and multidisciplinary study of cases of sudden cardiac death in the Czech Republic in the years 2016–2021, which was financed by a grant from the Ministry of Health of the Czech Republic with registration number NV18-02–00,237. The aim of the project was to identify a representative set of SCD cases. Subsequently, based on the interest of relatives and obtaining the informed consent of persons close to the deceased, find out the molecular causes of sudden heart death and evaluate the outcomes and impacts of this examination on the care of first-degree relatives for primary prevention of life-threatening heart rhythm disorders.

## Methods

This multidisciplinary and multi-center study has been approved by the Institute of Clinical and Experimental Medicine, the University Hospital Motol, and all participating Forensic Institutions Ethics Committee. Consent of post-mortem testing from all SCD cases included into the study was provided by close family members.

### Study cohort

From 2016 to 2021, we studied a cohort of 100 unrelated SCD victims and their families. Forensic department directly reported 61/100 cases suspected of dying from cardiovascular diseases, 19/100 cases were included based on family cardiologist recommendations, and 20/100 cases were added to the study based on family request. Forensic autopsy was performed in all included cases.

**Fig. 1:**
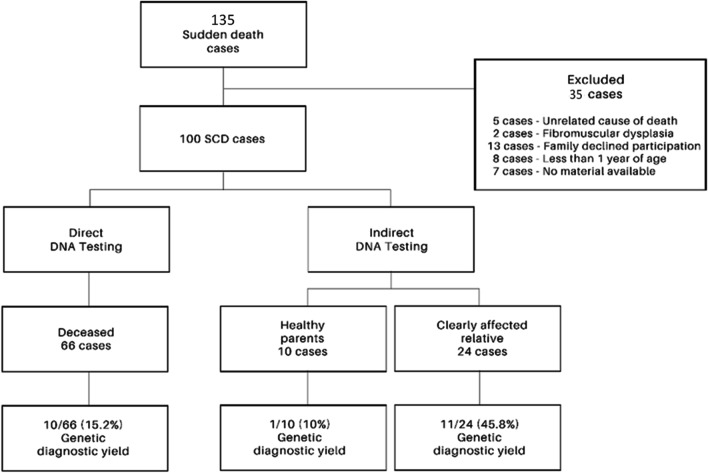
Study overview. Flowchart indicating the number of included/excluded cases into the study and cases who were identified by direct or indirect DNA genetic testing (i.e., DNA material origin) and corresponding genetic diagnostic yield (i.e., only P/LP variants detection). No material available indicates that we were not able to obtain DNA material from either deceased or their family members

Cases with a non-cardiovascular cause of death, lethal medications/toxins, age less than 1 year, where families declined participation in the study, and/or with CAD different from familial thoracic aortic aneurysm and dissection were excluded from the cohort. The study cohort flow is represented in Fig. 1.

**Fig. 2:**
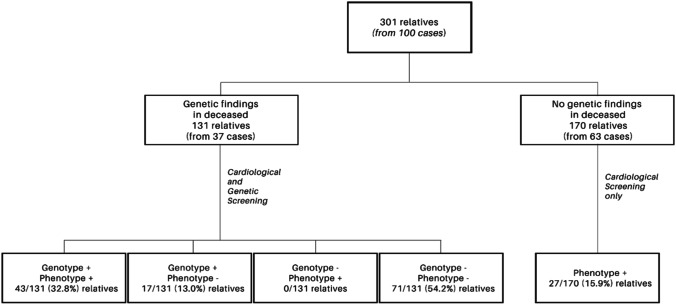
Family cascade screening. In SCD cases with genetic findings, family cardiological and genetic screening were performed. In SCD cases without genetic findings, only cardiological screening was offered to family members. Genetic findings include P/LP, VUS*, and RF variants. P/LP, pathogenic/likely pathogenic variant; VUS*, interesting variant of unknown significance; RF, risk factor

SCD victims aged between 1 and 59 were included into the study. Clinical data on the circumstances of death, health status, and family history were recorded (Table 1). Family testing was performed on 301 relatives of SCD victims. In 37/100 families where a variant of interest was detected, genetic testing in relatives at risk was performed. In families with a negative genetic test (63/100), only cardiological screening was performed (Fig. 2).

**Table 1:** Demographic and clinical data of the SCD study cohort. *CM*, cardiomyopathies; *SADS*, sudden arrhythmic death syndrome; *SUDS*, sudden unexpected death syndrome; *SAD*, sudden aortic death; *SD*, standard deviation; *SCD*, sudden cardiac death; *ICC*, inherited cardiac condition

	CM	SADS	SUDS	SAD	Total
No. of cases	49	20	23	8	100
Males	35/49 (71.4)	12/20 (60.0)	16/23 (69.6)	8/8 (100)	71/100 (71.0)
Females	14/49 (28.6)	8/20 (40.0)	7/23 (30.4)	-	29/100 (29.0)
Age at event, mean (SD), years					
Total cases	35.7 (11.8)	29.6 (13.1)	31.2 (14.3)	34.0 (12.0)	33.3 (12.8)
Males	36.4 (10.7)	23.6 (11.6)	28.6 (12.6)	34.0 (12.0)	32.2 (12.2)
Female	34.1 (14.5)	38.6 (9.9)	37.0 (17.4)	-	36.0 (13.8)
Place at event, *n*/*N* (%)					
Home	35/49 (71.4)	14/20 (70.0)	17/23 (73.9)	5/8 (62.5)	71/100 (71.0)
Work	3/49 (6.1)	1/20 (5.0)	1/23 (4.3)	-	5/100 (5.0)
Hospital bed wards	1/49 (2.0)	2/20 (10.0)	1/23 (4.3)	2/8 (25.0)	6/100 (6.0)
Public place	6/49 (12.2)	3/20 (15.0)	4/23 (17.4)	1/8 (12.5)	14/100 (14.0)
Unknown	4/49 (8.2)	-	-	-	4/100 (4.0)
Time at event, *n*/*N* (%)					
Day (6 am–10 pm)	22/49 (44.9)	9/20 (45.0)	9/23 (39.1)	1/8 (12.5)	41/100 (41.0)
Night (10 pm–6 am)	18/49 (36.7)	8/20 (40.0)	9/23 (39.1)	5/8 (62.5)	40/100 (40.0)
Unknown	9/49 (18.4)	3/20 (15.0)	5/23 (21.7)	2/8 (25.0)	19/100 (19.0)
Circumstances at event, *n*/*N* (%)					
Sleep	15/49 (30.6)	9/20 (45.0)	9/23 (39.1)	2/8 (25.0)	35/100 (35.0)
Rest	10/49 (20.4)	3/20 (15.0)	2/23 (8.7)	3/8 (37.5)	18/100 (18.0)
Light activities	12/49 (24.5)	5/20 (25.0)	9/23 (39.1)	2/8 (25.0)	28/100 (28.0)
Exertion/auditory/emotional stress	6/49 (12.2)	-	1/23 (4.3)	-	7/100 (7.0)
Unknown	6/49 (12.2)	3/20 (15.0)	2/23 (8.7)	1/8 (12.5)	12/100 (12.0)
Symptoms, *n*/*N* (%)					
Syncope	3/49 (6.1)	2/20 (10.0)	2/23 (8.7)	-	7/100 (7.0)
Seizures	2/49 (4.1)	2/20 (10.0)	2/23 (8.7)	1/8 (12.5)	7/100 (7.0)
Palpitations	5/49 (10.2)	2/20 (10.0)	2/23 (8.7)	1/8 (12.5)	10/100 (10.0)
Chest pain	6/49 (12.2)	2/20 (10.0)	1/23 (4.3)	2/8 (25.0)	11/100 (11.0)
Short of breath	9/49 (18.4)	3/20 (15.0)	2/23 (8.7)	1/8 (12.5)	15/100 (15.0)
Other/unknown	30/49 (61.2)	14/20 (70.0)	16/23 (69.6)	6/8 (75.0)	66/100 (66.0)
Family history of SCD and/or ICC, *n*/*N* (%)					
Yes	25/49 (51.0)	6/20 (30.0)	9/23 (39.1)	6/8 (75.0)	46/100 (46.0)
No	21/49 (42.9)	12/20 (60.0)	12/23 (52.2)	2/8 (25.0)	47/100 (47.0)
Unknown	3/49 (6.1)	2/20 (10.0)	2/23 (8.7)	-	7/100 (7.0)

### Autopsy evaluation

Autopsies of all SCD cases were performed at 12 different Czech Forensic Medicine Institutes from 8/13 regions of the Czech Republic. Post-mortem diagnosis was established by forensic autopsy, which included macroscopic and microscopic examination of the heart and blood vessels. All autopsies of the deceased were performed according to valid recommendations for the procedure of autopsy in the Czech Republic (Act No. 372/2011 Coll., on Health Services). In the course of the project cooperation, the expert group consisting of forensic pathologists, cardiologists, and cardiogeneticists was established in order to create Czech national autopsy guidelines based on European recommendations [8]. These are now in the approval process.

After forensic cardiac/aortic autopsy, cases were categorized in four major groups based on 2020 APHRS/HRS expert consensus statement [2]: (i) cardiomyopathies, cases with a confirmed diagnosis of heart structure; (ii) sudden arrhythmic death syndrome, unclear cause of death in an individual over 1 year of age with a negative pathological autopsy, i.e., without macroscopic, microscopic/necropsy, and toxicological findings; (iii) sudden unexplained death syndrome, unclear cause of death in an individual older than 1 year, when there are non-specific structural changes of the heart that do not meet the criteria for cardiomyopathy or arrhythmic syndrome, or necropsy was not performed; and (iv) sudden thoracic aortic death, cases with a confirmed diagnosis of aortic dissection leading to death. Characteristics of individual groups are described in Supplementary Table 2.

### Genetic testing

DNA testing for all samples was performed at the Genetic Department of University Hospital Motol. DNA post-mortem samples were obtained from tissue rich in nucleated cells collected during autopsy (i.e., spleen, nodules, or liver). Tissues prior DNA isolation were stored either in RNAlater solution for fresh tissue, frozen at − 20 °C or − 80 °C, or as formalin-fixed paraffin-embedded tissue. DNA samples for genetic testing in living family members were obtained from peripheral blood samples stored with K_3_EDTA.

Genomic DNA was extracted from tissue and/or blood samples using an automated nucleic acid extractor, MagCore HF16 Plus (RBC Bioscience, Taiwan). DNA was quantified using the NanoDrop 2000 spectrophotometer (Thermo Scientific, USA) and Qubit 2.0 (Invitrogen, USA) according to the manufacturer’s instructions. Next-generation sequencing (NGS) library preparation in all SCD cases was performed using either a broad custom-made panel comprising 100 cardiac/aortic conditions-related genes (Sophia Genetics, Switzerland). The full list of genes included in the custom-made panel is available in Supplementary Table 1. DNA libraries were sequenced by NGS with paired-end reads (2 × 150 bp cycles) on MiniSeq/MiSeq/NextSeq/NovaSeq platforms (Illumina, USA). NGS sequencing conditions used gave high coverage of all regions of interest allowing for copy number variation in all genes. In 24 negative cases, whole exome sequencing was performed. In 10/24 of these cases, the expanded analysis of two or three affected family members was performed due to a family history of SCD. All variants of interest were validated by Sanger DNA sequencing, and cascade family screening was performed. In the case of poor-quality DNA samples, NGS analysis was performed on clearly affected relatives in 24/100 cases or on healthy parents in 10/100 cases (Fig. 1). When a variant of interest was found by indirect DNA testing, it was confirmed by Sanger sequencing in the deceased.

### Data analysis

NGS sequencing data were processed and analyzed by Genome Analysis Toolkit pipeline from Broad Institute (USA). Variant calling was based on the human genome reference GRCh37/hg19. Variant prioritization was performed by Sophia DDM software supported by Integrative Genomics Viewer (Broad Institute), Alamut® Visual (Interactive Biosoftware), and VarSome Clinical software. Variant prioritization was carried out based on the presence and frequency of the variant in general population (gnomAD, dbSNP databases), presence in clinical databases (ClinVar, Human Gene Mutation Database), interspecies conservation of the residue and coherence, familial cosegregation with the phenotype and in silico predictions using bioinformatics tools integrated in Varsome Clinical software (DANN, DEOGEN2, EIGEN, FATHMM-MKL, M-CAP, MVP, MutationAssessor, MutationTaster, PrimateAI, REVEL, PolyPhen, and SIFT). Variants with read depth < 10 × , synonymous and intronic variants in non-splice regions, and minor allele frequency higher than expected for the disease were excluded [14]. The pathogenicity of the detected variants was classified into 5 categories according to the evidence criteria proposed by the American College of Medical Genetics and Genomics and the Association for Molecular Pathology (ACMG/AMP) guidelines [15]. P/LP variants are classified as class 4 and 5, VUS corresponds to class 3, and a common risk factor belongs to class 2. We have included an extra group of VUS*—VUS of interest—which are rare genetic variants of unknown significance located in known inherited cardiac/aortic condition genes with a high probability of being the disease cause based on current knowledge and molecular/clinical geneticists experience but lacking more substantial evidence such as functional studies and/or larger segregation studies.

## Results

### Demographic characteristics of the study cohort

In total, we have performed molecular autopsy in 100 unrelated SCD cases. The demographic characteristics of the study group are described in Table 1. SCD cases were represented mainly by males 71/100 (71.0%) with a mean age of death of 33.3 (12.8) and a range of 1 to 59 years. The cardiac event most often occurred while sleeping 35/100 (35.0%) and at home 71/100 (71.0%). Family history of inherited cardiovascular conditions and/or sudden death was reported in 46/100 (46.0%) of cases (Table 1).

### Molecular autopsy results

We performed molecular autopsy by next-generation sequencing. The diagnostic yield in this study was 22/100 (22.0%). From these 22 diagnosed cases, 23 P/LP variants were identified since we detected 2 P/LP variants in one case of cardiomyopathy (Tables 2 and 3). Two variants were transmitted de novo, and 21 were transmitted from their parents.

**Table 2:** Detailed overview of genetic findings. *CM*, cardiomyopathies; *SADS*, sudden arrhythmic death syndrome; *SUDS*, sudden unexpected death syndrome; *SAD*, sudden aortic death; *SD*, standard deviation; *P/LP*, pathogenic/likely pathogenic variant; VUS*, interesting variant of unknown significance; *RF*, risk factor; *FH* + , positive family history; *FH − *, negative family history; *SCD*, sudden cardiac death; *ICD*, inherited cardiac disease

Case	Genotype group	Autopsy group	CM subgroup	Gender	Age at death	Family history of SCD and/or ICD	DNA testing	Gene	OMIM (gene)	Associated disease	Inheritance	DNA variant	dbSNP	ACMG/AMP class	ACMG/AMP evidence
098	P/LP	CM	HCM	F	59	FH +	Indirect—affected relative	*GLA*	300,644	Fabry disease	XL	NM_000169.3(GLA):c.644A > G p.(Asn215Ser)	rs28935197	5	PM1, PM2, PP3, PP5
	P/LP							*KCNQ1*	607,542	Long QT syndrome 1	AD	NM_000218.2(KCNQ1):c.1781G > A p.(Arg594Gln)	rs199472815	5	PM1, PM2, PP3, PP5
064	P/LP	CM	HCM	M	47	FH +	Direct—proband	*MYBPC3*	600,958	HCM	AD	NM_000256.3(MYBPC3):c.1227-13G > A	rs397515893	4	PM2, PP3, PS1, PS3, PM2
008	P/LP	CM	ACM	M	36	FH +	Indirect—affected relative	*SCN5A*	600,163	DCM, LQTS, BrS	AD	NM_000335.5(SCN5A):c.4219G > A p.(Gly1407Arg)	rs137854612	5	PS1, PM2, PP3, PP1
057	P/LP	CM	DCM	F	37	FH −	Direct—proband	*RBM20*	613,171	DCM	AD	NM_001134363.3(RBM20):c.1900C > T p.(Arg634Trp)	rs796734066	5	PM1, PM2, PM5, PP3, PP5
068	P/LP	CM	HCM	M	16	FH +	Indirect—affected relative	*FHL1*	300,163	Emery-Dreifuss muscular dystrophy 6, X-linked	XLR	NM_001159699.2(FHL1):c.55G > T p.(Glu19*)	-	5	PVS1, PM2, PP3
020	P/LP	CM	DCM	M	27	FH −	Indirect—parents	*TTN*	188,840	DCM, ARVC	AD	NM_001267550.2(TTN):c.33172_33172 + 13del p.(Val11058fs)	-	4	PVS1, PM2
053	P/LP	CM	DCM	M	48	FH +	Indirect—affected relative	*TTN*	188,840	DCM, ARVC	AD	NM_001267550.2(TTN):c.34211_34212del p.(Tyr11404Cysfs*3)	-	4	PVS1, PM2
025	P/LP	CM	LVNC	M	35	FH +	Direct—proband	*TTN*	188,840	DCM, LVNC	AD	NM_001267550.2(TTN):c.44899C > T p.(Arg14967*)	rs727505350	5	PVS1, PM2, PP5
032	P/LP	CM	DCM	F	37	FH +	Indirect—affected relative	*TTN*	188,840	DCM, ARVC	AD	NM_001267550.2(TTN):c.46668_46671del p.(Glu15557Profs*28)	-	4	PVS1, PM2
096	P/LP	CM	DCM	M	45	FH +	Indirect—affected relative	*TTN*	188,840	DCM, ARVC	AD	NM_001267550.2(TTN):c.81124G > T p.(Glu27042*)	-	4	PVS1, PM2
028	P/LP	CM	ACM	M	26	FH −	Direct—proband	*FLNC*	102,565	Myopathy, myofibrillar	AD	NM_001458.5(FLNC):c.102G > A p.(Trp34*)	-	5	PVS1, PM2, PP3, PP5
051	P/LP	CM	DCM	M	34	FH +	Direct—proband	*FLNC*	102,565	Myopathy, myofibrillar	AD	NM_001458.5(FLNC):c.6406dup p.(Glu2136Glyfs*20)	-	5	PVS1, PM2, PP3
042	P/LP	CM	HCM	M	52	FH +	Indirect—affected relative	*MYPN*	608,517	HCM	AD	NM_032578.4(MYPN):c.3263G > A p.(Arg1088His)	rs71584501	4	PM2, PP5, PP1
050	P/LP	SAD	-	M	16	FH +	Direct—proband	*COL3A1*	120,180	Ehlers-Danlos syndrome, vascular type	De novo	NM_000090.4(COL3A1):c.1330G > C p.(Gly444Arg)	rs587779489	5	PS1, PS2, PM1, PM2
099	P/LP	SAD	-	M	16	FH +	Direct—proband	*COL3A1*	120,180	Ehlers-Danlos syndrome, vascular type	AD	NM_000090.4(COL3A1):c.2654G > A p.(Gly885Asp)	-	4	PM1, PM2, PP2, PP3
012	P/LP	SAD	-	M	35	FH +	Indirect—affected relative	*TGFBR1*	190,181	Loeys-Dietz syndrome	AD	NM_004612.4(TGFBR1):c.1460G > A p.(Arg487Gln)	rs113605875	5	PM1, PM2, PM5, PP2, PP3, PP5
021	P/LP	SADS	-	F	41	FH +	Indirect—affected relative	*KCNH2*	152,427	LQT2	AD	NM_000238.4(KCNH2):c.43del p.(Leu15Trpfs*45)	-	5	PVS1, PM2, PP1
044	P/LP	SADS	-	M	4	FH −	Direct—proband	*RYR2*	180,902	CPVT	De novo	NM_001035.3(RYR2):c.7202G > A p.(Arg2401His)	rs794728756	5	PS2, PM2, PM5,PP3, PP5
065	P/LP	SUDS	-	F	36	FH +	Indirect—affected relative	*RYR2*	180,902	CPVT	AD	NM_001035.3(RYR2):c.1197G > A p.(Met399Ile)	rs727503397	4	PM1 PM2, PP1, PP3
088	P/LP	SUDS	-	F	54	FH +	Indirect—affected relative	*TTN*	188,840	DCM, ARVC	AD	NM_001267550.2(TTN):c.103360del p.(Glu34454Asnfs*3)	rs760768093	5	PVS1, PM2, PP5
089	P/LP	SUDS	-	M	38	FH +	Direct—proband	*TNNT2*	191,045	HCM, DCM	AD	NM_001276345.2(TNNT2):c.40G > T p.(Glu14*)	rs772890125	5	PVS1, PM2, PP3
090	P/LP	SUDS	-	M	13	FH −	Direct—proband	*FLNC*	102,565	Myopathy, myofibrillar	AD	NM_001458.5(FLNC):c.3070C > T p.(Gln1024*)	-	5	PVS1, PM2, PP3
035	VUS*	CM	HCM	M	19	FH −	Direct—proband	*MYH7*	160,760	HCM	AD	NM_000257.4 (MYH7):c.2908G > T p.(Ala970Ser)	rs1446657813	3	PM2, PP2
003	VUS*	CM	ACM	F	24	FH −	Indirect—parents	*SCNA5*	600,163	DCM, LQTS, BrS	AD	NM_000335.5(SCN5A):c.246C > A p.(Asp82Glu)	rs747643709	3	PM2, PP2, PP3
071	VUS*	CM	ACM	M	37	FH −	Direct—proband	*SCN5A*	600,163	DCM, LQTS, BrS	AD	NM_000335.5(SCN5A):c.4886 T > C p.(Ile1629Thr)	-	3	PM2, PP3
077	VUS*	CM	ACM	M	53	FH −	Direct—proband	*DES*	125,660	DCM, ARVC	AD	NM_001927.4(DES):c.641A > C p.(Asp214Ala)	-	3	PM2, PP2, PP3
038	VUS*	CM	DCM	M	39	FH +	Indirect—affected relative	*DES*	125,660	DCM, ARVC	AD	NM_001927.4(DES):c.976C > T p.(His326Tyr)	rs794728987	3	PM2, PP2, PP3
061	VUS*	CM	ACM	F	20	FH +	Direct—proband	*DSP*	125,647	DCM, ARVC	AD	NM_004415.4(DSP):c.4558A > G p.(Ser1520Gly)	rs780953141	3	PM2, PP3
007	VUS*	CM	ACM	M	17	FH −	Direct—proband	*CTNNA3*	607,667	ARVC	AD	NM_013266.4(CTNNA3):c.592C > A p.(Pro198Thr)	rs777817815	3	PM2, BP4
004	VUS*	CM	HCM	M	36	FH −	Direct—proband	*PRKAG2*	602,743	Glycogen storage disease	AD	NM_016203.4(PRKAG2):c.593dupC p.(Asp199Glyfs*74)	rs397517275	3	PM2, PP3
037	VUS*	SADS	-	M	26	FH −	Direct—proband	*DPP6*	152,427	LQT	AD	NM_130797.4(DPP6):c.2350 T > A p.(Phe784Ile)	rs1238655957	3	PM2, PP3
001	VUS*	CM	ACM	F	28	FH +	Direct—proband	*LMNA*	150,330	DCM	AD	NM_170707.4(LMNA):c.1660G > A p.(Glu554Lys)	rs71630616	3	PM2, PP2, BP4
	RF							*KCNE1*	176,261	LQT5 lite	AD	NM_000219.6(KCNE1):c.253G > A p.(Asp85Asn)	rs1805128	2	BS1
011	RF	CM	DCM	M	33	FH −	Direct—proband	*KCNE1*	176,261	LQT5 lite	AD	NM_000219.6(KCNE1):c.253G > A p.(Asp85Asn)	rs1805128	2	BS1
026	RF	SADS	-	M	6	FH −	Direct—proband	*KCNE1*	176,261	LQT5 lite	AD	NM_000219.6(KCNE1):c.253G > A p.(Asp85Asn)	rs1805128	2	BS1
022	RF	SADS	-	F	41	FH +	Indirect—affected relative	*KCNE1*	176,261	LQT5 lite	AD	NM_000219.6(KCNE1):c.253G > A p.(Asp85Asn)	rs1805128	2	BS1
019	RF	SUDS	-	M	15	FH −	Direct—proband	*KCNE1*	176,261	LQT5 lite	AD	NM_000219.6(KCNE1):c.253G > A p.(Asp85Asn)	rs1805128	2	BS1

**Table 3:** P/LP variants detection. Diagnostic genetic yield reached by each autopsy diagnosis group. *P/LP*, pathogenic/likely pathogenic; *CM*, cardiomyopathies; *SADS*, sudden arrhythmic death syndrome; *SUDS*, sudden unexpected death syndrome; *SAD*, sudden aortic death

Genetic yield	CM	SADS	SUDS	SAD	Total
P/LP findings	13/49 (26.5)	2/20 (10.0)	4/23 (17.4)	3/8 (37.5)	22/100 (22.0)
No significant findings	36/49 (73.5)	18/20 (90.0)	19/23 (82.6)	5/8 (62.5)	78/100 (78.0)

We have identified 10/100 (10.0%) of VUS* in genes known to be disease-causing but lacking strong evidence to be in the P/LP category (Table 2).

The autopsy group with the highest P/LP variant detection rate was the SAD group 3/8 (37.5%), followed by CM 13/49 (26.5%) (Table 3). Within the CM group, the highest diagnostic genetic yield was from cases related to DCM 6/14 (42.9%). The lowest P/LP variant detection rate observed was from the post-mortem arrhythmogenic cardiomyopathy (ACM) group 2/22 (9.1%) (Table 4).

**Table 4:** P/LP variants detection. Detailed diagnostic yield by CM forensic diagnosis cases. *P/LP*, pathogenic/likely pathogenic; *HCM*, hypertrophic cardiomyopathy; *DCM*, dilated cardiomyopathy; *LVNC*, left ventricular non-compaction; *ACM*, arrhythmogenic cardiomyopathy

Genetic yield	HCM	DCM	ACM	LVNC
P/LP findings	4/12 (33.3)	6/14 (42.9)	2/22 (9.1)	1/1 (100)
No significant findings	8/12 (66.7)	8/14 (57.1)	20/22 (90.9)	-

**Fig. 3:**
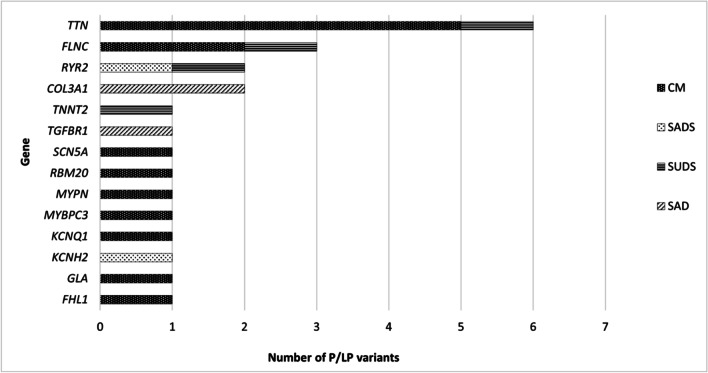
List of genes and occurrence of P/LP variants detected. P/LP, pathogenic/likely pathogenic; CM, cardiomyopathies; SADS, sudden arrhythmic death syndrome; SUDS, sudden unexpected death syndrome; SAD, sudden aortic death

The most frequently altered gene detected was *TTN*, and variants in this gene were from cases diagnosed as DCM, LVNC, and SUDS accounting for 6/23 (26,1%) of the P/LP variants detected (Fig. 3). We have also identified P/LP variants in HCM phenocopy genes such as *GLA* related to Fabry disease and *FHL1* related to X-linked muscular dystrophy (Table 2). No pathogenic copy number variations (CNV) were detected.

In 10 clearly familiar SCD with negative results on targeted panels, the expanded analysis with other affected family members was performed (whole exome sequencing, Sophia Genetics, Switzerland) without yielding new P/LP DNA variants.

We have also identified a known DNA risk factor variant (class 2, likely benign) in the potassium channel gene: NM_000219.5(KCNE1): c.253G > A p.(Asp85Asn). This variant is not rare in the normal population (total MAF:0.009324, non-Finnish European MAF:0.01223, gnomAD) and is a risk allele for drug-induced long QT syndrome type 5, with a mild course and incomplete penetrance [14]. The detailed overview of genetic findings is in Table 2.

**Fig. 4:**
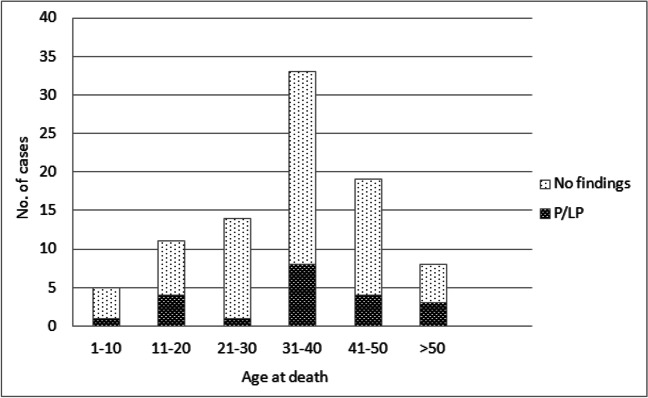
Distribution of P/LP variants by age at death groups. P/LP, pathogenic/likely pathogenic

The highest P/LP variant detection rate 8 of 36 (22.2%) was identified in the age group of 31 to 40 years at death (Fig. 4).

### Direct and indirect DNA testing

Due to poor quality DNA, in 34/100 (34.0%) SCD cases, NGS analysis was indirectly performed on a clearly affected relative or on both healthy parents (Fig. 1). It was due to the unavailability of material other than formalin-fixed and paraffin-embedded tissue and, in several cases, tissue autolysis prior to DNA extraction. At the time of this study, EDTA blood sampling was not routinely performed at autopsy by forensic specialists.

**Fig. 5:**
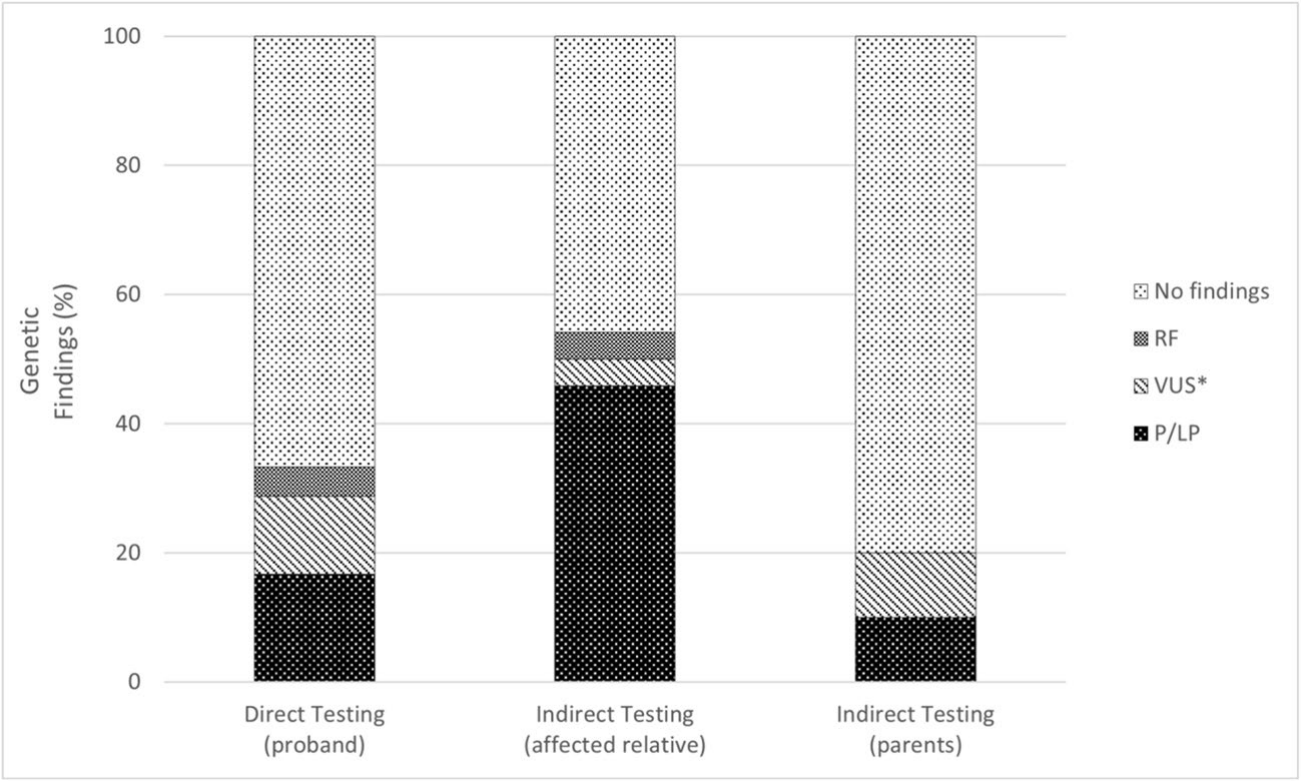
Genetic findings (i.e., P/LP, VUS*, RF variants identification). Identification of variants of interest according to DNA testing type: direct in proband’s sample; indirect in an affected family member or indirect in healthy parents. P/LP, pathogenic/likely pathogenic variant; VUS*, interesting variant of unknown significance; RF, risk factor

**Fig. 6:**
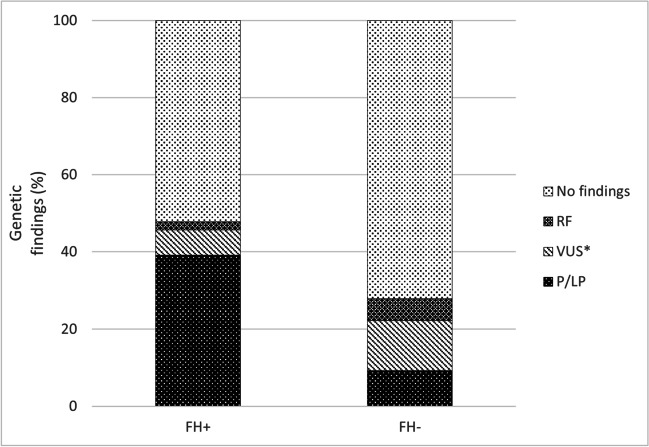
Family history and percentage of genetic findings (i.e., P/LP, VUS*, RF variants identification). P/LP, pathogenic/likely pathogenic variant; VUS*, interesting variant of unknown significance; RF, risk factor; FH + , positive family history; FH − , negative family history

Indirect testing in affected family members had a diagnostic yield of 11/24 (45.8%), while genetic testing performed in healthy parents reached a diagnostic yield of 1/10 (10.0%) (Figs. 1 and 5). In cases with family history of SCD and/or inherited cardiac condition (ICC), we have identified 18/46 (39.0%) P/LP variants, while in SCD cases without family history, we have identified 5/54 (9.0%) causative variants (Fig. 6, Table 2).

### Family screening

**Fig. 7:**
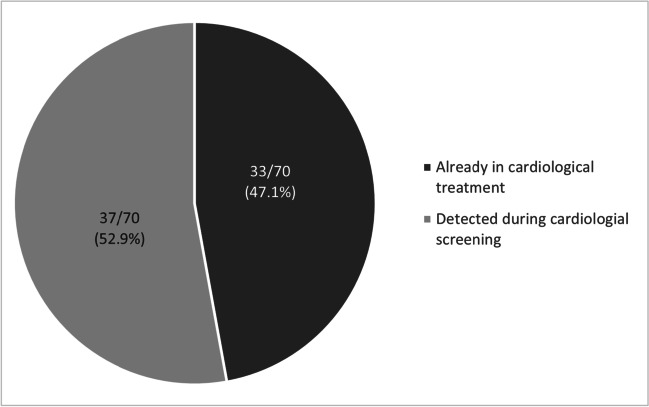
Phenotype-positive family members and disease awareness. This chart represents the proportion of phenotype positive (independent of genetic–genetic findings) family members diagnosed with an ICC and time of diagnosis. Half patients were either already in cardiological care at the time to be included into the family screening program or they were diagnosed for the first time during our family management scheme

A total of 301 relatives from 100 families were examined, of whom 87/301 (28.9%) had a positive cardiological phenotype and/or a positive genotype (Fig. 2). Genetic screening for identified DNA variants (P/LP, VUS*, and RF variants) was performed in 37 families, in 131 relatives. A positive genetic finding was found in 60/131 (45.8%) of relatives. Through cardiological family screening, we uncovered 70 affected individuals with an ICC (phenotype-positive family members); 33/70 (47.1%) were already in cardiological treatment before SCD of their first-degree relative, and 37/70 (52.9%) were newly diagnosed through our family cascade screening (Fig. 7).

## Discussion

We present the results of an unprecedented post-mortem genetic study performed in the Czech Republic. Our study substantially contributed to establishing a multidisciplinary collaboration on a national level. Through post-mortem molecular genetic analysis, we identified pathogenic/likely pathogenic variants (P/LP) following ACMG/AMP recommendations in 22/100 (22.0%) of cases. Cardiological and genetic screening disclosed 83/301 (27.6%) relatives at risk of SCD. Genetic testing in affected relatives as starting material leads to a high diagnostic yield (45,8%) and offers a valuable alternative when suitable material is not available in our study.

The vast majority of Czech families (122/135, 90.4%) are interested in investigating the causes of death of their relatives and in preventive cardiological care as documented (Fig. 1).

Most of the SCD cases were males (71.0%), corresponding to known gender differences in the severity of cardiomyopathies [16, 17]. The higher incidence of SCD in males has been previously reported [18, 19].

Most sudden deaths occurred at home and during daily routine activity or sleep, consistent with international studies [3, 20]. Only 7% of deaths occurred during vigorous sport or physical activity. Sudden death in an athlete was not reported to us during the study time (Table 1).

The mean age of studied cases was less than 40 years (33.3 years), while the males with post-mortem diagnosis SADS were the youngest (23.6 years) (Table 1) as described elsewhere [21].

The overall genetic yield of 22.0% observed in our study is consistent with published international studies [21–23]. Genetic testing findings highly correlate with the autopsy diagnosis in all groups.

Thus, in general, genetic testing can be expected to identify clearly inherited conditions in about 1/5 of SCD cases. The yield and spectrum of detected variants in our SCD study correspond to that observed in other European cohorts [19, 21–23].

The only surprising result was low recovery in the ACM group, although all ACM-related genes were tested [24] (Supplementary Table 1). The European autopsy guidelines are not yet fully adopted in the Czech Republic, and the autopsy diagnosis of ACM may not be correct in some cases. Thanks to the ongoing cooperation and multidisciplinary communication, we believe to overcome this burden soon.

The extended NGS panels did not bring a higher diagnostic yield even in clearly familiar cases in consistency with other studies [25].

These results of genetic yield in our cohort are crucial for communication with family members so that their expectations for this type of testing are realistic.

We have confirmed the high increase in the genetic diagnostic yield (P/LP) (39%) in cases with family history of SCD and/or ICC whereas in cases without family history, the diagnostic yield was only 9% (Fig. 6). The effect of positive FH is reflected in high diagnostic yield (i.e., P/LP only variants) obtained through genetic testing in affected family members in cases where quality DNA was not available for testing and comprises even a 45.8% (Figs. 1 and 5).

This shows the importance of detailed cardiological screening in surviving relatives bringing the possibility of genetic testing in affected individuals.

We did not find copy-number variants in our SCD cohort, but Sophia Genetics enables their detection, and we indeed detect them in cases outside this study.

The frequent titin (*TTN*) pathogenic findings reflect the known high frequency in familiar heart failure and its arrhythmogenic potential and highly correlate with the autopsy findings (Table 2, Fig. 3) [26, 27]. Titin is a giant myofilament that extends from the Z-disk (N-terminus) to the M-band (C-terminus) region of the sarcomere and is now recognized as a major human disease gene. Many titin mutations are linked to cardiomyopathies and neuromuscular diseases [28].

In our study, we identified truncating variants in the filamin C gene (*FLNC*) as a certain molecular cause in three male individuals from the DCM, ACM, and SUDS groups. FLNC is the gene encoding filamin C, an actin cross-linking protein that plays a central role in the assembly and organization of sarcomeres. Gene is widely expressed in cardiac and skeletal muscles, and mutations in FLNC were associated with skeletal myopathy, as well as hypertrophic, restrictive, and dilated cardiomyopathy [29]. The heterogeneous autopsy findings correlate with the described clinical manifestations of the *FLNC* gene and also show that arrhythmic complications may precede the development of clear structural changes in the heart muscle [25–28]. In this study, one family with a P/LP variant in *FLNC* gene, requested to be included in an assisted reproduction and preimplantation diagnosis program for primary prevention of the disease in offspring [30–33].

The inclusion of HCM-phenocopy genes in the NGS genetic panel (Supplementary Table 1), allowed the identification of P/LP variants in the *GLA*, *PRKAG2*, and *FHL1* genes, increasing the overall genetic diagnostic detection rate.

The finding of a common RF variant in the potassium channel gene *KCNE1* associated with hereditary arrhythmia syndrome LQT5 lite is difficult to interpret in the deceased. Based on the available literature, we did not identify it as a clear molecular cause of sudden death [34–36]. In 4 out of 5 families, the LQT5 lite variant segregated along with the cardiological phenotype (QTc prolongation) and complaints. Nevertheless, we communicated the finding to families and recommended appropriate medicament treatment and lifestyle measurements [37].

Detected variants of unknown significance (VUS) are a challenge for clinical interpretation [38, 39]. Nevertheless, we decided to assign the class of VUS* variants (Table 2). These variants are rare in population and located in genes related to inherited cardiovascular diseases, prediction software and practice knowledge supports a pathogenic role, and they segregate with the phenotype within the family. In these families, we informed the relatives about the finding and offered all of them cardiological follow-up every 3–5 years; nevertheless, VUS* carriers are included in more intensive preventive diagnostic programs.

We see a great advantage in the centralization of molecular genetic testing, whereby we have ensured uniform methodological procedures and, finally, the assessment of identified DNA variants with the standardized assignment of the corresponding diagnostic criteria according to the ACMG/AMP recommendations, which may otherwise differ among laboratories in molecular genetic practice.

Our results lead to primary prevention of SCD in almost 1/3 of relatives at risk, with most of them in productive age. In families with detected genetic variant (P/LP, VUS*, RF), the proportion of relatives at potential risk is even higher (45.8%) (Fig. 2). Half of family relatives have already been treated for ICC. Genetic testing contributed so to their accurate diagnosis. Nevertheless, the other family members were not aware of their present health condition with the risk of SCD. These findings should contribute to the higher awareness in caring professionals of the possibility of underlying genetic background (i.e., familial disease) in heart diseases and encourage them to offer the family cascade screening. Our findings further document that the genetic yield is in familiar cases even higher than in the general cohort. The molecular genetic analysis in affected relatives may be used for molecular autopsy if the material from the SCD victims is not of good quality. Nevertheless, the screening in genetic negative cases identified 15.9% phenotype-positive relatives, and so the clinical examination in this group should be recommended.

Our project has significantly improved the communication and collaboration among clinical genetics, molecular genetics, cardiology, and forensic medicine centers in several regions of the Czech Republic. Based on this study, the multidisciplinary teams are now being created in most national tertiary medical centers. The study founded the nationwide registry of SCD cases. Our project initiated the creation of Czech national guidelines on autopsy in the case of SCD which are now in the approval process.

## Conclusion

The post-mortem genetic analysis in the Czech Republic is feasible and of interest to included professionals and affected families. The diagnostic genetic yield is corresponding to other international cohorts. The family cascade screening should be offered to surviving relatives as recommended elsewhere. In clearly clinically familiar cases, the genetic yield is expected to be higher than in sporadic cases. The awareness of the possibility of familiar diseases should be increased in caring cardiologists, who should offer the family cascade screening in patients’ families with unexplained heart failure and/or ventricular arrhythmias. This established the basis for organized post-mortem analysis with necessary multidisciplinary teams at national and regional tertiary centers.

## Limitations

The main limitation was the difference in the description of the section findings among referring forensic centers since in the CZ, there were no established standard guidelines to perform the autopsy in suspected SCD cases. We dealt with the not exactly defined post-mortem diagnoses and the lack of material sufficient for molecular genetic testing. The post-mortem macroscopic and microscopic findings were often individually discussed with the forensic specialists and their classification to the internationally acknowledged categories as SADS, SUDS, or CM was assigned later after tedious discussion and after long intervals after death.

Another challenge we have encountered is the low diagnostic yield of 9.1% observed in cases of arrhythmogenic cardiomyopathy, highly discordant from studies observed in living adult patients with a reported diagnostic yield of 30–60% [40]. This last might be due to post-mortem overdiagnosis of ACM for cases presenting a fatty heart. To obtain data on body composition from an autopsy case is highly difficult in our experience and might be considered when an arrhythmogenic cardiomyopathy case is suspected.

### Supplementary Information

Below is the link to the electronic supplementary material.

Supplementary file1 (DOCX 11 KB)

Supplementary file2 (DOCX 12 KB)

Supplementary file3 (DOCX 14 KB)

## References

[1] Goldstein S (1982). The necessity of a uniform definition of sudden coronary death: witnessed death within 1 hour of the onset of acute symptoms. Am Heart J.

[2] Stiles MK, Wilde AAM, Abrams DJ (2021). 2020 APHRS/HRS expert consensus statement on the investigation of decedents with sudden unexplained death and patients with sudden cardiac arrest, and of their families. Heart Rhythm.

[3] Wong CX, Brown A, Lau DH (2019). Epidemiology of sudden cardiac death: global and regional perspectives. Heart Lung Circ.

[4] Rücklová K, Dobiáš M, Bílek M (2022). Burden of sudden cardiac death in persons aged 1–40 years in the Czech Republic. Cent Eur J Public Health..

[5] Charron P, Arad M, Arbustini E (2010). Genetic counselling and testing in cardiomyopathies: a position statement of the European Society of Cardiology Working Group on Myocardial and Pericardial Diseases. Eur Heart J.

[6] Zeppenfeld K, Tfelt-Hansen J, de Riva M et al (2022) ESC Guidelines for the management of patients with ventricular arrhythmias and the prevention of sudden cardiac death. Eur Heart J 43(40):3997–4126. 10.1093/eurheartj/ehac262 10.1093/eurheartj/ehac262 36017572

[7] Basso C, Burke M, Fornes P (2008). Guidelines for autopsy investigation of sudden cardiac death. Virchows Arch.

[8] Basso C, Aguilera B, Banner J (2017). Guidelines for autopsy investigation of sudden cardiac death: 2017 update from the Association for European Cardiovascular Pathology. Virchows Arch.

[9] Verhagen JMA, Kempers M, Cozijnsen L (2018). Expert consensus recommendations on the cardiogenetic care for patients with thoracic aortic disease and their first-degree relatives. Int J Cardiol.

[10] Harris SL, Lubitz SA (2020). Clinical and genetic evaluation after sudden cardiac arrest. J Cardiovasc Electrophysiol.

[11] Coll M, Oliva A, Grassi S, Brugada R, Campuzano O (2019) Update on the genetic basis of sudden unexpected death in epilepsy. Int J Mol Sci 20(8):1979. 10.3390/ijms20081979 10.3390/ijms20081979 PMC6515014 31018519

[12] Semsarian C, Ingles J, Wilde AAM (2015). Sudden cardiac death in the young: the molecular autopsy and a practical approach to surviving relatives. Eur Heart J.

[13] Fellmann F, van El CG, Charron P (2019). European recommendations integrating genetic testing into multidisciplinary management of sudden cardiac death. Eur J Hum Genet.

[14] Whiffin N, Minikel E, Walsh R (2017). Using high-resolution variant frequencies to empower clinical genome interpretation. Genet Med.

[15] Richards S, Aziz N, Bale S (2015). Standards and guidelines for the interpretation of sequence variants: a joint consensus recommendation of the American College of Medical Genetics and Genomics and the Association for Molecular Pathology. Genet Med.

[16] Butters A, Arnott C, Sweeting J, Winkel BG, Semsarian C, Ingles J (2021) Sex disparities in sudden cardiac death. Circ Arrhythm Electrophysiol 14(8):e009834. 10.1161/CIRCEP.121.009834 10.1161/CIRCEP.121.009834 34397259

[17] Kim SK, Bennett R, Ingles J, Kumar S, Zaman S (2021). Arrhythmia in cardiomyopathy: sex and gender differences. Curr Heart Fail Rep.

[18] Kong MH, Fonarow GC, Peterson ED (2011). Systematic review of the incidence of sudden cardiac death in the United States. J Am Coll Cardiol.

[19] Larsen MK, Christiansen SL, Hertz CL (2020). Targeted molecular genetic testing in young sudden cardiac death victims from Western Denmark. Int J Legal Med.

[20] Thakur RK, Hoffmann RG, Olson DW (1996). Circadian variation in sudden cardiac death: effects of age, sex, and initial cardiac rhythm. Ann Emerg Med.

[21] Lahrouchi N, Raju H, Lodder EM (2017). Utility of post-mortem genetic testing in cases of sudden arrhythmic death syndrome. J Am Coll Cardiol.

[22] Lahrouchi N, Raju H, Lodder EM (2020). The yield of postmortem genetic testing in sudden death cases with structural findings at autopsy. Eur J Hum Genet.

[23] Raju H, Parsons S, Thompson TN (2019). Insights into sudden cardiac death: exploring the potential relevance of non-diagnostic autopsy findings. Eur Heart J.

[24] Wilde AA, Semsarian C, Márquez MF (2022). European Heart Rhythm Association (EHRA)/Heart Rhythm Society (HRS)/Asia Pacific Heart Rhythm Society (APHRS)/Latin American Heart Rhythm Society (LAHRS) expert consensus statement on the state of genetic testing for cardiac diseases. Heart Rhythm Published online.

[25] Marschall C, Moscu-Gregor A, Klein HG (2019) Variant panorama in 1,385 index patients and sensitivity of expanded next-generation sequencing panels in arrhythmogenic disorders. Cardiovasc Diagn Ther 9(Suppl 2):S292–S298. 10.21037/CDT.2019.06.06 10.21037/cdt.2019.06.06 PMC6837920 31737537

[26] Akhtar M, Elliott PM. (2019) Risk stratification for sudden cardiac death in non-ischaemic dilated cardiomyopathy. 10.1007/s11886-019-1236-3 10.1007/s11886-019-1236-3 PMC6877704 31768884

[27] Mazzarotto F, Tayal U, Buchan RJ (2020). Reevaluating the genetic contribution of monogenic dilated cardiomyopathy. Circulation.

[28] Kellermayer D, Smith JE, Granzier H (2019). Titin mutations and muscle disease. Pflugers Arch - Eur J Physiol.

[29] Begay RL, Graw SL, Sinagra G (2018). Filamin C Truncation mutations are associated with arrhythmogenic dilated cardiomyopathy and changes in the cell–cell adhesion structures. JACC Clin Electrophysiol.

[30] Isbister JC, Nowak N, Butters A (2021). “Concealed cardiomyopathy” as a cause of previously unexplained sudden cardiac arrest. Int J Cardiol.

[31] Verdonschot JAJ, Vanhoutte EK, Claes GRF (2020). A mutation update for the FLNC gene in myopathies and cardiomyopathies. Hum Mutat.

[32] Ortiz-Genga MF, Cuenca S, Dal Ferro M (2016). Truncating FLNC mutations are associated with high-risk dilated and arrhythmogenic cardiomyopathies. J Am Coll Cardiol.

[33] Ader F, de Groote P, Réant P (2019). FLNC pathogenic variants in patients with cardiomyopathies: prevalence and genotype-phenotype correlations. Clin Genet.

[34] Garmany R, Giudicessi JR, Ye D, Zhou W, Tester DJ, Ackerman MJ (2020). Clinical and functional reappraisal of alleged type 5 long QT syndrome: causative genetic variants in the KCNE1-encoded minK β-subunit. Heart Rhythm.

[35] Roberts JD, Asaki SY, Mazzanti A et al (2020) An international multicenter evaluation of type 5 long QT syndrome: a low penetrant primary arrhythmic condition. Circulation 141(6):429–439. 10.1161/CIRCULATIONAHA.119.043114 10.1161/CIRCULATIONAHA.119.043114 PMC7035205 31941373

[36] Lane CM, Giudicessi JR, Ye D (2018). Long QT syndrome type 5-Lite: Defining the clinical phenotype associated with the potentially proarrhythmic p.Asp85Asn-KCNE1 common genetic variant. Heart Rhythm..

[37] Wilde AAM, Amin AS, Postema PG (2022). Diagnosis, management and therapeutic strategies for congenital long QT syndrome. Heart.

[38] Grassi S, Campuzano O, Coll M (2020). Genetic variants of uncertain significance: how to match scientific rigour and standard of proof in sudden cardiac death?. Leg Med (Tokyo).

[39] Campuzano O, Sarquella-Brugada G, Fernandez-Falgueras A et al (2020) Reanalysis and reclassification of rare genetic variants associated with inherited arrhythmogenic syndromes. BioMedicine 54:102732. 10.1016/j.ebiom.2020.102732 10.1016/j.ebiom.2020.102732 PMC7136601 32268277

[40] Gandjbakhch E, Redheuil A, Pousset F, Charron P, Frank R (2018). Clinical diagnosis, imaging, and genetics of arrhythmogenic right ventricular cardiomyopathy/dysplasia: JACC state-of-the-art review. J Am Coll Cardiol.

