# Supplementary material for: Post-mortem genetic testing in sudden cardiac death and genetic screening of relatives at risk: lessons learned from a Czech pilot multidisciplinary study
Source: Int J Legal Med. 2023 May 13;137(6):1787–801. doi: 10.1007/s00414-023-03007-z (PMC10567875; doi:10.1007/s00414-023-03007-z)
Supplement: Supplementary file 1 — Supplementary file1 (DOCX 11 KB) [file 414_2023_3007_MOESM1_ESM.docx]

SuppTab. 1: Custom-made panel comprising 100 cardiac/aortic conditions-related genes.

| Cardiomypathies | Arrhythmias | Aortopaties | Congenital heart diseases |
| --- | --- | --- | --- |
| *ACTC1, ACTN2, BAG3, CSRP3, CTNNA3, DES, DSC2, DSG2, DSP, DTNA, FHL1, FLNA, FLNC, GLA, LAMP2, LDB3, LMNA, MIB1, MYBPC3, MYH6, MYH7, MYL2, MYL3, MYPN, NEXN, PKP2, PLN, PRKAG2, PTPN11, RAF1, RBM20, SGCD, TCAP, TMEM43, TNNC1, TNNI3, TNNT2, TPM1, TTN, VCL* | *ANK2, CACNA1C, CALM1, CALM2, CASQ2, CAV3, KCNA5, KCNE1, KCNE2, KCNH2, KCNJ2, KCNJ5, KCNQ1, SCN5A, SNTA1, TRDN* | *ACTA2, COL1A1, COL1A2, COL3A1, COL4A3, COL4A4, COL4A5, COL5A1, COL5A2, ELN, EMILIN1, FBN1, FBN2, LOX, MAT2A, MFAP5, MYH11, NOTCH1, PLOD1, SKI, SLC2A10, SMAD3, SMAD4, SMAD6, TGFB2, TGFB3, TGFBR1, TGFBR2, TNXB* | *ACTA2, ELN, GATA4, GATA5, GATA6, JAG1, NKX2.5, NOTCH1, NOTCH2, PTPN11, TBX1, TBX20, TBX5, ZIC3* |
