# Supplementary material for: Post-mortem genetic testing in sudden cardiac death and genetic screening of relatives at risk: lessons learned from a Czech pilot multidisciplinary study
Source: Int J Legal Med. 2023 May 13;137(6):1787–801. doi: 10.1007/s00414-023-03007-z (PMC10567875; doi:10.1007/s00414-023-03007-z)
Supplement: Supplementary file 2 — Supplementary file2 (DOCX 12 KB) [file 414_2023_3007_MOESM2_ESM.docx]

SuppTab. 2: Characteristics of individual SCD groups

| **cardiomyopathies (CM)** | **sudden arrhythmic death syndrome (SADS)** | **sudden unexplained death syndrome (SUDS)** | **sudden thoracic aortic death (SAD)** |
| --- | --- | --- | --- |
| cases with a confirmed diagnosis of heart structure | unclear cause of death in an individual over 1 year of age with a negative pathological autopsy i.e. without macroscopic, microscopic / necropsy and toxicological findings | unclear cause of death in an individual older than 1 year, when there are non-specific structural changes of the heart that do not meet the criteria for CM or AS, or necropsy was not performed and | cases with a confirmed diagnosis of aortic dissection leading to death. |
