# Supplementary material for: Post-mortem genetic testing in sudden cardiac death and genetic screening of relatives at risk: lessons learned from a Czech pilot multidisciplinary study
Source: Int J Legal Med. 2023 May 13;137(6):1787–801. doi: 10.1007/s00414-023-03007-z (PMC10567875; doi:10.1007/s00414-023-03007-z)
Supplement: Supplementary file 3 — Supplementary file3 (DOCX 14 KB) [file 414_2023_3007_MOESM3_ESM.docx]

| **Abbreviations** | **Definition** |
| --- | --- |
| SCD | sudden cardiac death |
| CM | cardiomyopathy |
| HCM | hypertrophic cardiomyopathy |
| ACM | arrhythmogenic cardiomyopathy |
| DCM | dilated cardiomyopathy |
| LVNC | left ventricular noncompaction cardiomyopathy |
| SADS | sudden arrhythmic death syndrome |
| SUD | sudden unexplained death |
| SUDS | sudden unexplained death syndrome |
| SUDI | sudden unexplained deaths in infant |
| SUDEP | sudden unexplained death in epilepsy |
| SAD | sudden aortic death |
| CAD | coronary artery disease |
| QTc | Corrected QT Interval |
| DNA | deoxyribonucleic acid |
| EDTA | ethylenediaminetetraacetic acid |
| NGS | next-generation sequencing |
| CNV | copy number variant |
| GATK | genome Analysis Toolkit |
| WES | whole exome sequencing |
| ACMG/AMP | American College of Medical Genetics and Genomics and the Association for Molecular Pathology |
| RF | risk factor |
| VUS | variant of uncertain significance |
| P/LP | pathogenic/likely pathogenic |
| APHRS / HRS | Asia Pacific Heart Rhythm Society / Heart Rhythm Society |
| AECVP | Association for European Cardiovascular Pathology |

SuppTab. 3: List of abbreviations
